# Tree Alignment Based on Needleman-Wunsch Algorithm for Sensor Selection in Smart Homes

**DOI:** 10.3390/s17081902

**Published:** 2017-08-18

**Authors:** Sook-Ling Chua, Lee Kien Foo

**Affiliations:** Faculty of Computing and Informatics, Multimedia University, Persiaran Multimedia, 63100 Cyberjaya, Selangor, Malaysia; slchua@mmu.edu.my

**Keywords:** sensor selection, tree alignment, needleman-wunsch algorithm, activity recognition, smart homes

## Abstract

Activity recognition in smart homes aims to infer the particular activities of the inhabitant, the aim being to monitor their activities and identify any abnormalities, especially for those living alone. In order for a smart home to support its inhabitant, the recognition system needs to learn from observations acquired through sensors. One question that often arises is which sensors are useful and how many sensors are required to accurately recognise the inhabitant’s activities? Many wrapper methods have been proposed and remain one of the popular evaluators for sensor selection due to its superior accuracy performance. However, they are prohibitively slow during the evaluation process and may run into the risk of overfitting due to the extent of the search. Motivated by this characteristic, this paper attempts to reduce the cost of the evaluation process and overfitting through tree alignment. The performance of our method is evaluated on two public datasets obtained in two distinct smart home environments.

## 1. Introduction

Activity recognition has drawn significant attention from the machine learning research community due to its growing demand from many potential applications such as security and surveillance (e.g., detecting suspicious activities in airports), industrial applications (e.g., monitoring of activities performed by workers on assembly lines), healthcare (e.g., monitoring patient’s disease progression), and sports (e.g., monitoring the quality of execution), amongst others.

One application of activity recognition that supports people in their daily activities is the smart home. The smart home has gained popularity due to its role in supporting the inhabitants, typically older adults who are living alone. Low-powered, unobtrusive sensors such as state-change sensors, motion sensors, pressure mats, etc. are commonly used to capture information about the inhabitant. These sensors are attached to the household objects in the home (e.g., a television, cupboard, etc.) and are activated when the inhabitant performs their daily activities. For example, turning on or off the bathroom light would activate the sensor attached to it. Sensor data collected from the smart home is used by the activity recognition system to learn and monitor the inhabitant’s daily activities.

Many supervised and unsupervised methods have been proposed for activity recognition [1,2,3]. These methods attempt to learn from as many sensors as possible with the aim that the classifier acquires a good representation of the inhabitant’s activities. Unfortunately, training on a bank of sensors not only requires more training data but also has an effect on recognition performance. The challenge, however, is to identify which sensors are useful and how many sensors are required to effectively recognise the activities of the inhabitant.

**Figure 1:**
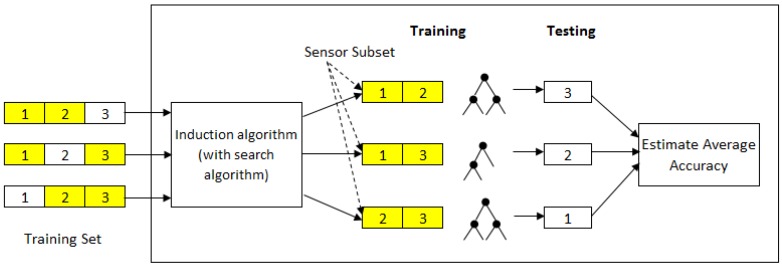
Wrapper-based sensor selection using 3-fold cross-validation.

The wrapper method is one of the commonly used methods for sensor selection. It uses an induction algorithm (e.g., a decision tree) as an evaluation function to score the sensors based on their predictive performance. It aims to find a subset of sensors in such a way that when an induction algorithm is trained on this reduced set of sensors, it will produce a classifier with better recognition accuracy. A greedy sequential search algorithm is often applied to search through the space of possible sensors. Depending on the search, it can begin with either an empty set of sensors or a full set of sensors. The former is called forward selection, while the latter is called backward elimination. To reduce variability resulting from the data, multiple rounds of cross-validation are performed using different partitions of the training set. The validation results are then averaged over the rounds. This means that for each sensor subset that is evaluated, an induction algorithm is invoked *k*-times in a *k*-fold cross-validation. Such an approach, however, is computationally expensive as a new classifier has to be trained for each subset evaluation. Figure 1 shows an example of wrapper-based sensor selection using 3-fold cross-validation.

A search algorithm that is dependent on accuracy estimates may choose a sensor subset with high accuracy but poor predictive power [4]. Such a method, which is guided by accuracy estimates, may result in overfitting at the expense of generalisation to a previously unseen sensor subset. This motivates us to look into methods to address the sensor selection problem without the need to rely on any search algorithm and accuracy estimates. In this paper, we take a different approach to sensor selection. Rather than sequentially evaluate each sensor subset, we train the decision tree directly on each partition of the training set and then address the sensor generalisation through tree alignment. We demonstrate our approach on two public datasets obtained in two distinct smart home environments.

The paper is organised as follows. Section 2 discusses related works on sensor selection. A discussion of our proposed method is presented in Section 3. Section 4 details the real world data used and the evaluation method. Section 5 discusses the experimental results. Section 6 presents the discussion regarding the improvement of our method over previous work. Finally, we summarise the work presented in this paper.

## 2. Related Work

Both filter-based and wrapper-based methods are widely used to select informative sensors for activity recognition. The filter-based approach relies on some heuristics to evaluate the characteristic of the sensors. In the works of both Chahuara et al. [5], and Chua and Foo [6], information gain criterion were used to select the set of informative sensors. Classifiers were trained on sensors with non-zero information gain. Cook and Holder [7] used the mutual information criterion to measure dependency between sensors and activities. Sensors with high mutual information are considered as informative since they can best discriminate between activities. Similar work was also seen in Dobrucali and Barshan [8], where they used mutual information criterion on wearable motion sensors to select the set of informative sensors based on sensor types, measurement axes, and sensor locations. The filter-based approach for sensor selection may not necessarily reduce the number of sensors. These sensors are usually ranked based on the their importance and there is a need to rely on prior knowledge to define a suitable cut-off point in order to determine the number of sensors needed in the final subset.

The wrapper-based approach uses an induction algorithm to score sensors based on their predictive performance. Bourobou et al. [2] used the decision tree as a learning algorithm for sensor selection. Attal et al. [9] applied the random forest as a wrapper method to identify the set of informative sensors for recognising human physical activities, such as sitting, lying, standing, etc. Saputri, Khan, and Lee [10] used a three-stage process based on a genetic algorithm for finding common sensors of physical activity for each subject. Mafrur et al. [11] used the support vector machine with sequential floating forward selection to reduce both loading and prediction time for activity recognition on mobile phones. However, the wrapper-based approach is more computationally expensive than the filter-based approach and may run the risk of overfitting [4,12].

Some methods attempt to reduce overfitting by using an early-stopping strategy, i.e., to stop the search before overfitting occurs. Verachtert et al. [13] applied a dynamic stopping condition into naïve Bayes. They used support and accuracy criteria to dynamically determine the stopping point. These criteria are estimated from a validation set, which means that the validation set needs to acquire a good data representation in order to accurately determine a suitable threshold. The work by Loughrey and Cunnigham [14] proposed a genetic algorithm with an early-stopping mechanism through the use of nested cross-validation. Inner cross-validation is used to tune the parameters, while outer cross-validation evaluates the accuracy of the training and validation sets. However, the parameters of the genetic algorithm are dependent on the dataset being used.

## 3. Our Proposed Method

Our approach to the sensor selection problem is to train a decision tree directly on each partition of the training set. Tree alignment is then performed on the trained trees to find a tree where the similarity between this tree and all given trees is maximal. Tree alignment is a way of arranging the sequences in trees to identify regions of similarity among them.

**Figure 2:**
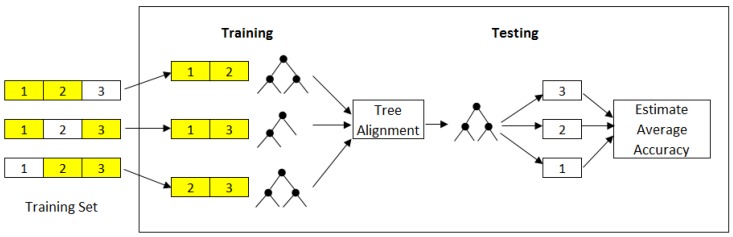
Illustration of our proposed method for sensor selection. Tree building is first performed on each training set. Then, tree alignment is performed to find a tree such that the similarity between this tree and all other trees is maximal.

Figure 2 shows our proposed method based on tree alignment for sensor selection, which contrasts to prior works given it eliminates the need for testing the decision tree models for each subset evaluation. To measure the similarity between pairs of trees, we used the Needleman-Wunsch algorithm [15].

### Needleman-Wunsch Algorithm

The Needleman-Wunsch algorithm [15] is commonly used as a global alignment technique in bio-informatics to align protein or nucleotide sequences. The algorithm uses a scoring system by giving a value for each match, mismatch, and indel (gap). If a match is +1, mismatch −1, and gap −1, the alignment score for two sequences, x= ‘ACTGA’ and y= ‘ACAA’ is 1. The alignment for sequences *x* and *y* is as follows:
ACTGA||

|AC–AA

Sequences may have different lengths (seen in the case of the sequences *x* and *y*), which is the reason why letters are paired up with dashes in the other sequence, to signify either insertions or deletions in the sequences.

**Figure 3:**
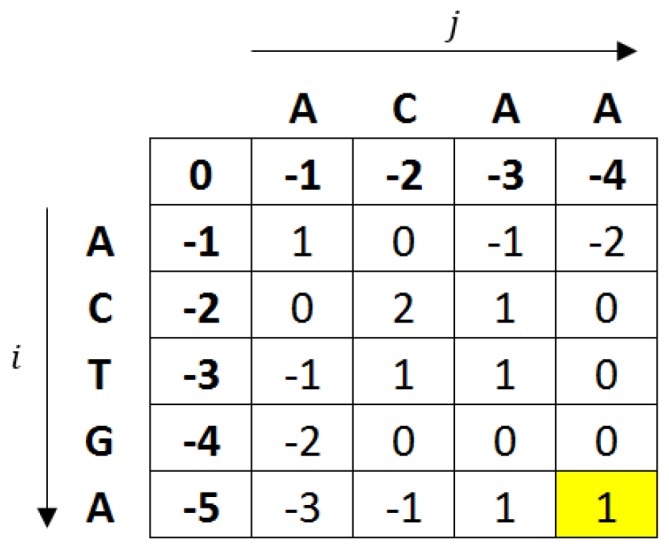
An illustration of how the Needleman-Wunsch algorithm can be computed using dynamic programming to align the sequences ‘ACTGA’ and ‘ACAA’. The score in each cell is computed using Equation (1), with match +1, mismatch −1, and a gap −1.

The Needleman-Wunsch algorithm uses a two-dimensional matrix (size (|x|+1)×(|y|+1), where |x| and |y| are the lengths of the sequences *x* and *y*) to keep track of the alignment score. The algorithm first initialises the first column and first row as 0 and subsequently adds the gap score for the first column, i.e., having values [0,−1,−2,…,|x|] and, similarly, for the first row, to have values [0,−1,−2,…,|y|]. The score for each remaining cell is computed using the following Equation (1):

(1) $$M_{i,j}=\max\left[M_{i-1,j-1}+s_{i,j},M_{i,j-1}+g,M_{i-1,j}+g\right]$$

where $M_{i,j}$ is the element of the *i*th row and *j*th column in the *M* matrix, $s_{i,j}$ is the substitution score (i.e., $s_{i,j}=1$ if the letter at position *i* is the same as the letter at position *j*, and $s_{i,j}=-1$ if there is a mismatch), and *g* is the gap penalty. The value on the last row and column in the matrix (shaded in Figure 3) represents the alignment score.

**Figure 4:**
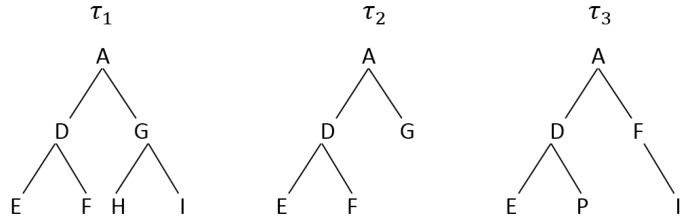
Example showing three distinct trees with different lengths and sequences.

Based on the Needleman-Wunsch algorithm, similarity between a pair of trees can be computed. A high alignment score indicates that two trees are similar. To show this, we use the example of the trees $\tau_{1}$, $\tau_{2}$, and $\tau_{3}$, shown in Figure 4. A pre-order tree traversal is first performed on the trees. This generates a sequence of ‘ADEFGHI’ for $\tau_{1}$, ‘ADEFG’ for $\tau_{2}$, and ‘ADEPFI’ for $\tau_{3}$. A tree alignment is then performed on the generated sequences using the Needleman-Wunsch algorithm. The alignment score of $\tau_{1}$ and $\tau_{2}$ is 3 while $\tau_{1}$ and $\tau_{3}$ have a score of 2, which clearly shows that $\tau_{1}$ is more similar to $\tau_{2}$ than $\tau_{3}$. This process is repeated for every pair of trees. The tree $\tau_{max}$, with the highest average similarity score, is chosen as the ‘best’ tree whereby all the sensors in that tree are considered as informative.

Algorithm 1 shows the steps of using Needleman-Wunsch algorithm for tree alignment. The output is a score matrix containing the alignment score for every pair of trees. This matrix is then used to calculate the average similarity score.

**Algorithm 1** Tree Alignment Using Needleman-Wunsch Algorithm**Require:** a set of trees $\tau_{1},\tau_{2},\ldots ,\tau_{k}$ **Ensure:**
i=1 **Ensure:**
$x_{seq}\leftarrow$ Perform pre-order tree traversal on $\tau_{i}$ **while** not end of *k*
**do**  **for**
j=1tok
**do**   **if**
i≠j
**then**    $y_{seq}\leftarrow$ Perform pre-order tree traversal on $\tau_{j}$    $Score_{i,j}\leftarrow$ Using Equation (1), calculate similarity between $x_{seq}$ and $y_{seq}$   **end if**   i=i+1  **end for** **end while** $\tau_{max}\leftarrow$ Select tree with max of average similarity score of Score matrix

Finding similarity in tree data structures has been used extensively in XML documents [16,17]. The Levenshtein edit distance [18] is commonly used as a measure of similarity to transform one tree into another by applying edit operations such as insertion, deletion, and substitution. The main difference between the Needleman-Wunsch algorithm and the Levenshtein distance algorithm is that the Levenshtein distance algorithm used a static penalty cost to any mismatched letters whilst the Needleman-Wunsch algorithm gives weights to matches and mismatches differently.

## 4. Datasets and Evaluation Method

To demonstrate the efficacy of our proposed method, we used two distinct smart home datasets—MIT PlaceLab [19] and van Kasteren [20].

### 4.1. MIT PlaceLab Dataset

The first dataset is obtained from the MIT PlaceLab [19]. They used a total of 77 state-change sensors to capture the activities of the inhabitant living inside. The subject kept a record of his activities, meaning that there was a ground truth annotation of the dataset. The data was collected for a period of 16 days. In this study, our interest was in recognising the activity of daily living and thus we did not consider objects that were rarely used. In view of this, we only consider sensors that were activated more than 20 times throughout the 16 day period, resulting in a total of 24 sensors in this dataset.

A total of six activities was identified from this set of 24 sensors, which were grooming/dressing, preparing meal/beverages, washing/putting away dishes, toileting/showering, doing/putting away laundry, and cleaning. The number of activity examples used in this dataset is shown in Table 1a.

**Figure 5:**
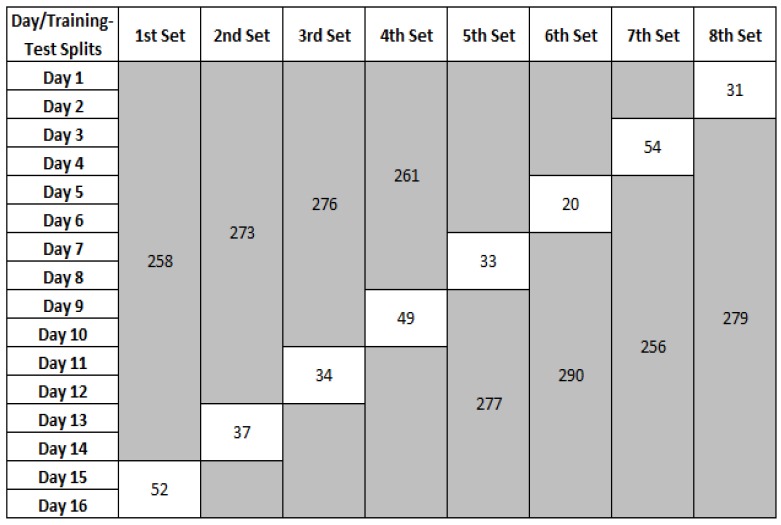
Leave-two days-out cross-validation on MIT PlaceLab dataset. Training sets are shaded in grey and the unshaded are testing sets. The values in each of the training-test splits refer to the number of activity examples in the training and test sets, respectively.

For each evaluation, we used a leave-two days-out cross-validation method to calculate the confusion matrix. From the total of 16 days, we used 14 days for training and the remaining two days for testing. The main reason for using two days for testing is to ensure that every activity is seen in the test set since some activities such as ‘washing dishes’, ‘cleaning’, and ‘doing laundry’ do not occur daily. The process is repeated eight times. Figure 5 shows the leave-two days-out cross validation method on the MIT PlaceLab dataset along with the number of activity examples used in each training and test set.

### 4.2. Van Kasteren Dataset

**Figure 6:**
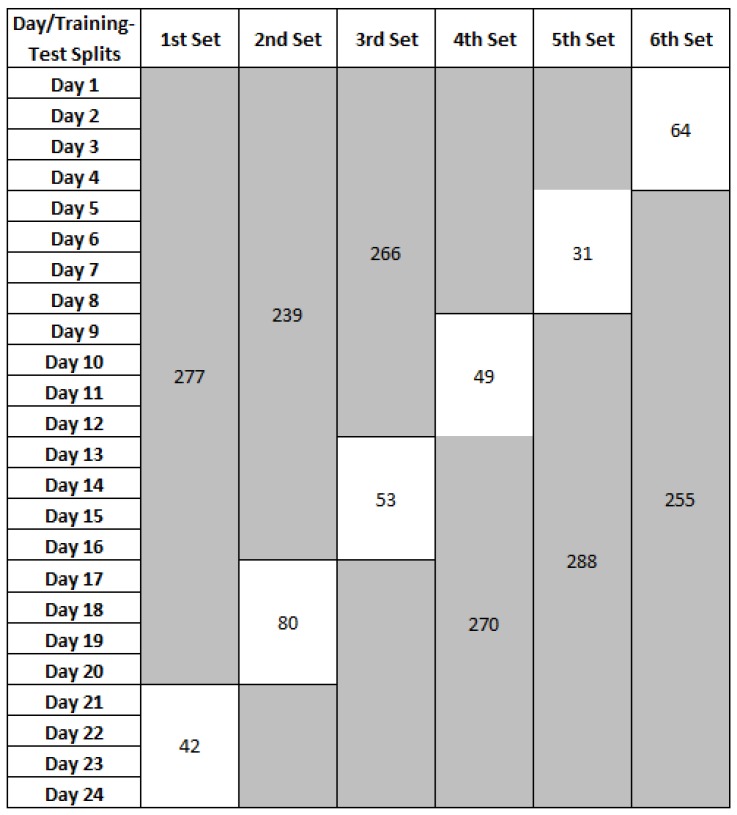
Leave-four days-out cross-validation on van Kasteren dataset. Training sets are shaded in grey and the unshaded are the testing sets. The values in each of the training-test splits refer to the number of activity examples in the training and test sets, respectively.

The second dataset is obtained from van Kasteren [20]. A total of 14 state-change sensors were used to collect information about the occupant living in a three-room apartment. The data was collected over a period of 24 days. There are four activities in this dataset—leave house, toileting/showering, go to bed, and preparing meal/beverages. The number of activity examples used in this dataset is shown in Table 1b. Since the number of activities that occur each day is relatively small in this dataset, we used 20 days for training and the remaining four days for testing. The process was repeated six times. Figure 6 shows the leave-four days-out cross-validation method applied on van Kasteren dataset.

To measure the performance of the inferred activities against user activities, we used four performance measures—recognition accuracy, precision, recall, and F-measure (F1):

(2) $$\begin{array}{cc} Accuracy= & \sum_{i=1}^{N}\frac{TP_{i}}{TP_{i}+TN_{i}+FP_{i}+FN_{i}} \end{array}\;$$

(3) $$\begin{array}{cc} \;Precision= & \frac{1}{N}\sum_{i=1}^{N}\frac{TP_{i}}{TP_{i}+FP_{i}} \end{array}$$

(4) $$\begin{array}{cc} Recall= & \frac{1}{N}\sum_{i=1}^{N}\frac{TP_{i}}{TP_{i}+FN_{i}} \end{array}$$

(5) $$\begin{array}{cc} \;F1= & 2\cdot \frac{Precision\cdot Recall}{Precision+Recall} \end{array}$$

where *N* is the number of activities, $TP_{i}$ is the number of true positives for activity *i*, $FP_{i}$ is the number of false positives for activity *i*, $TN_{i}$ is the number of true negatives for activity *i* and $FN_{i}$ is the number of false negatives for activity *i*. Recognition accuracy is the proportion of true positives over the total number of activities examined. Precision measures the percentage of inferred activities correctly recognised while recall measures the percentage of ground truth activities correctly recognised. F1 calculates the harmonic mean of precision and recall. The precision and recall are calculated for each activity separately. Since some activities appear much more frequently than other activities, we take the average precision and recall over all activities and consider the correct recognition of each activity as equally important. The final recognition performance is calculated by averaging the accuracies in each evaluation.

In all the experiments conducted, we trained on four different classifiers, i.e., decision tree, naïve Bayes classifier, linear discriminant analysis, and *k*-nearest neighbors. These four classifiers were trained with the purpose of validating the results of sensor selection, not to determine which classifier gives the best recognition rate.

## 5. Experiments and Results

We conducted three experiments. In the first experiment, we trained on the sensors selected through the tree alignment by applying the Needleman-Wunch algorithm. We then compared the result to the full set of baseline sensors to see how effective the proposed method is. In the second experiment, we compared our method with two baseline methods, while in the third we looked at the computational performance.

### 5.1. Experiment 1: Sensor Selection Based on Tree Alignment

In this experiment, we first trained the decision tree on each training set of the MIT PlaceLab dataset (discussed in Section 4). This results in a total of eight trees. From these trained trees, we performed tree alignment using the Needleman-Wunch algorithm. In our work, we used −2 for a gap, −1 for an unmatched, and +2 for a matched. The reason for setting a higher gap penalty is to reduce the overall score caused by insertions or deletions in sequences. These values, however, are determined empirically. Table 2 shows the results.

Referring to the table, $\tau_{6}$ has the highest average similarity score. This means that $\tau_{6}$ is more similar to all the other trees. Thus, all sensors in this tree are considered as informative. There are a total of 13 sensors in $\tau_{6}$. To test how well this set of 13 sensors recognises the activities of the inhabitant, we removed the other 11 sensors from the training and test sets respectively. The rationale of removing these sensors is as though they were removed physically from the home [7]. We then trained the four classifiers on the 13 sensors.

We repeated the same procedure on the van Kasteren dataset. In this dataset, $\tau_{1},\tau_{2}$, and $\tau_{5}$ each have the same similarity score. Further investigations showed that these trees have identified the same set of six sensors. It was observed that there are less activity variations in this dataset (compared to MIT PlaceLab), which resulted in tree resemblance. The classifiers are trained on all the six informative sensors (the other eight sensors were removed from the training and test sets, respectively).

**Figure 7:**
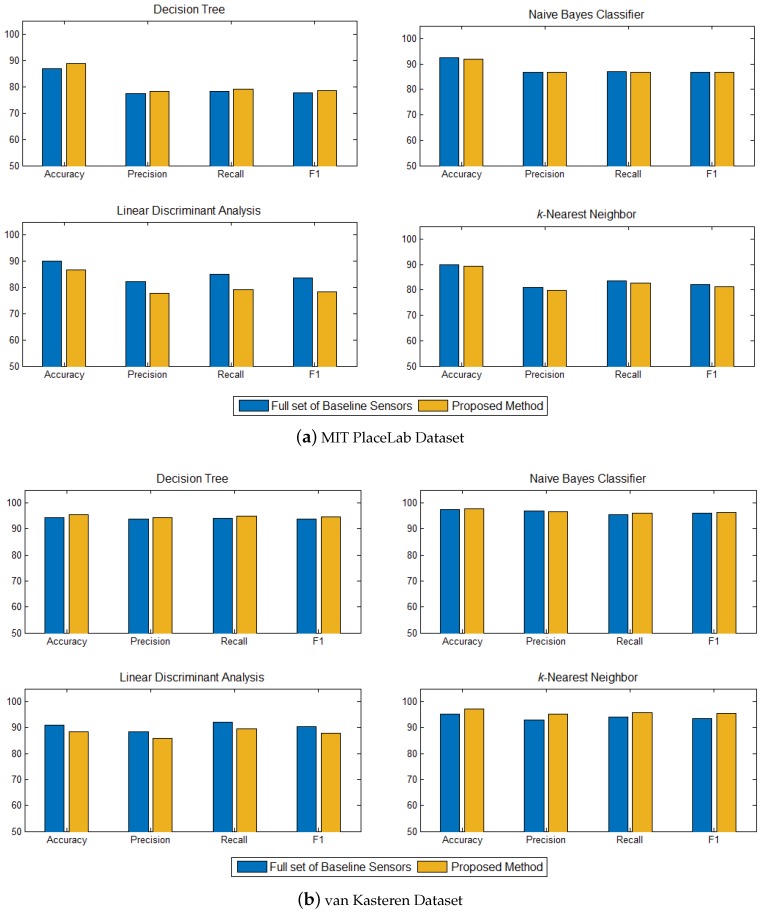
Average recognition performance of the proposed method with the full set of baseline sensors for (**a**) MIT PlaceLab dataset and (**b**) van Kasterenn dataset on four classifiers—decision tree, naïve Bayes classifier, linear discriminant analysis and *k*-nearest neighbor.

We also compared the set of informative sensors identified with the full set of baseline sensors to see how effective the proposed method is. For the MIT PlaceLab, we trained the four classifiers on the full set of 24 sensors and 14 sensors on the van Kasteren datasets. The results are shown in Figure 7.

In comparison to the full set of 24 sensors on MIT PlaceLab dataset (Figure 7a), our proposed method, which was based on 13 informative sensors, performed better on the decision tree but not as good in the linear discriminant analysis. However, the differences in both cases are not significant. The accuracies of our method obtained on naïve Bayes and *k*-nearest neighbor are comparable to the full set of 24 sensors.

As for the van Kasteren dataset (Figure 7b), our method, which trained on six informative sensors achieved almost the same accuracy with the full set of 14 sensors across all the classifiers. The encouraging results on both datasets have shown that the proposed method works effectively to identify the set of informative sensors for activity recognition.

### 5.2. Experiment 2: Comparison with the Baseline Methods

In this experiment, we compared our proposed method with two baseline methods—a naive approach and a wrapper method with sequential forward selection.

#### 5.2.1. A Naive Approach for Sensor Selection

The first baseline method is to select a tree that best classifies the activities by cross-validation. This is indeed a naive approach with the assumption that the tree that best classifies the activities consists of sensors that are informative. In this experiment, we first trained the decision tree on each training set and then tested the performance of the learned tree on the test sets. The tree with the highest average recognition accuracy is selected as the best classification tree. Table 3 shows the results on both datasets.

Referring to Table 3a, $\tau_{7}$ has the highest average recognition accuracy and thus identified as the ‘best’ tree for the MIT PlaceLab dataset. In this tree, there is a total of 16 informative sensors. As for the van Kasteren dataset (Table 3b), $\tau_{1}$ and $\tau_{5}$ achieved the same recognition accuracy. Further investigations on these trees showed that they have identified the same set of sensors. From the full set of 14 sensors, these trees have identified six sensors as informative.

**Figure 8:**
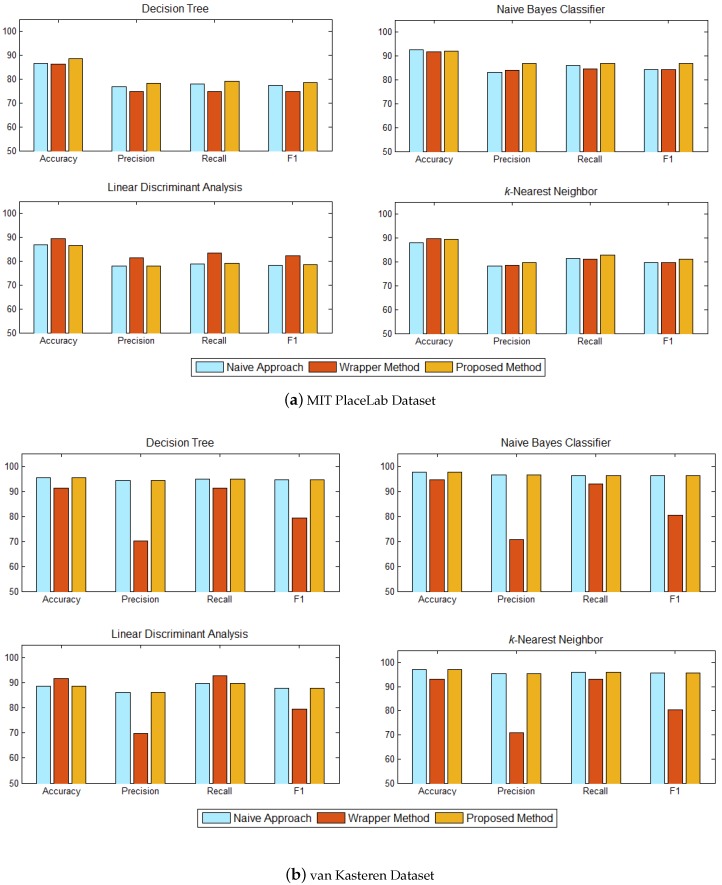
Recognition performance between our proposed method with baseline methods—(1) Naive approach and (2) Wrapper method with sequential forward selection in terms of accuracy, precision, recall, and F1. Each subplot shows the performance of each classifier—decision tree, naïve Bayes classifier, linear discriminant analysis, and *k*-nearest neighbor.

Once the ‘best’ classification tree had been identified, we then trained the four classifiers on the set of informative sensors, i.e., 16 sensors on the MIT PlaceLab dataset and six sensors on the van Kasteren dataset. The results are shown in Figure 8.

#### 5.2.2. Wrapper with Sequential Forward Selection

For the second baseline method, we used the sequential forward selection method and linear discriminant analysis as the learning algorithm. Sequential forward selection is a greedy search algorithm that sequentially select sensors that best predict activities until there is no improvement in prediction.

For the MIT PlaceLab dataset, 20 out of 24 sensors were selected as informative while seven out of 14 sensors were selected from the van Kasteren dataset. We then trained the classifiers on the set of selected informative sensors.

The recognition performance between our proposed method and the baseline methods are shown in Figure 8. Each subplot shows the performance of each classifier—decision tree, naïve Bayes classifier, linear discriminant analysis, and *k*-nearest neighbor. For the MIT PlaceLab, our method achieved almost the same accuracies as the baseline wrapper method across all the classifiers, did slightly better in decision tree and not as good in the linear discriminant analysis. The baseline wrapper method has the lowest precision and F1 across all the classifiers on the van Kasteran dataset. This method also has a lower accuracy and recall in the decision tree, naïve Bayes classifier, and *k*-nearest neighbor.

### 5.3. Experiment 3: Computational Performance

In this experiment, we looked at the computational performance between our method and baseline methods. To evaluate the overall computational performance, 30 runs on each test set were carried out on both datasets. Table 4 shows the average computation time (in sec) between our method and the baseline methods on each test set.

Our method has a lower running time compared to the two baseline methods on the van Kasteran dataset. There is no difference in running time between our method and the naive approach on the MIT dataset but the baseline wrapper method takes a longer time to run.

## 6. Discussion

Table 5 shows the total number of informative sensors identified for each method. Our method identified a smaller subset of informative sensors compared to the baseline methods for both datasets. As for the van Kasteren dataset, both our method and the baseline naive approach identified the same set of sensors.

For the MIT PlaceLab dataset (Figure 8a), our method achieved an accuracy comparable to the two baseline methods across all the classifiers. Although our proposed method does not appear to be significantly better, it used only 13 informative sensors to recognise the inhabitant’s activities, while the baseline naive method used 16 sensors and the baseline wrapper method used 20 sensors. In comparison with the baseline naive method, our method has a higher precision, recall, and F1 across all the classifiers, which shows that our method is able to identify the set of informative sensors that is better suited for activity recognition. Among all the classifiers, the baseline wrapper method has a higher recognition performance when trained on the linear discriminant analysis. This is expected as we used the linear discriminant analysis as the learning algorithm to select sensors for the baseline wrapper method.

As for the van Kasteren dataset (Figure 8b), as both of our method and the baseline naive method identified the same set of sensors, they achieved the same recognition performance across all the classifiers and significantly better than the baseline wrapper method. In terms of accuracy and recall, the baseline wrapper method had a better recognition for the linear discriminant analysis since the learning algorithm used for the wrapper method is trained using the linear discriminant analysis. However, the baseline wrapper method has the lowest precision and F1 across all the classifiers, which means that many activities have been incorrectly classified. The performance of our method achieved a consistent performance in accuracy, precision, recall, and F1 across all the classifiers, which makes our method suitable for sensor selection.

In terms of computational performance (see Table 4), the baseline wrapper method has a longer running and evaluation time since such a method requires a new classifier to be trained on each sensor subset evaluation. Although the difference is not significant in magnitude, the baseline wrapper method takes at least 50 folds longer (2.53 s as compared to the 0.05 s of our method) to run on the first set of the MIT PlaceLab dataset. When the number of sensors for evaluation increased from 14 (van Kasteren) to 24 (MIT PlaceLab), the computational time, on average, increased by 40%. This is expected as when the number of sensors for evaluation increases, a larger sensor space needs to be examined and thus takes a longer time to evaluate. The additional computational cost for the wrapper method is definitely non-trivial if we are performing sensor selection on a larger dataset.

The wrapper method in general uses cross-validation to guide the search through the use of validation sets to assess the predictive ability of the learning algorithm over the sensor subset. Such a method, which is guided by accuracy estimates, may result in overfitting. As can be seen from Figure 8, the wrapper method, overall, achieved better recognition accuracy but lower precision, recall, and F1 across decision tree, naïve Bayes classifier, and *k*-nearest neighbour, on both datasets. Since our method does not rely on a search algorithm nor does it depend on any accuracy estimates, it can help to reduce overfitting and have a better ability to generalise. Referring to Figure 8, our method has better precision, recall, and F1 on both datasets, which shows that our method is able to identify the set of sensors that is well suited for activity recognition.

## 7. Conclusions

In this paper, we propose a method that addresses the generalisability of sensors among multiple decision trees based on tree alignment for sensor selection in smart homes. We have evaluated our method compared to two baseline methods (i.e., a naive approach and a wrapper with sequential forward selection) on two distinct smart home datasets. We have also compared our method with the full set of baseline sensors. Results showed that our method can effectively identify the set of informative sensors for activity recognition. Our method outperformed the baseline methods on both datasets and is comparable to the full set of sensors used for activity recognition. In terms of computational time, our method has a shorter running and evaluation time compared to the baseline methods. Addressing the sensor selection problem not only helps to reduce the number of arbitrary sensors needed but also to improve recognition performance. We plan to extend our work by testing it on other datasets and domains.

## Figures and Tables

**Table 1:** Number of activity examples for each activity.

Activity	No of Activity Examples
(a) MIT PlaceLab Dataset	
Grooming/dressing	67
Preparing meal/beverages	79
Washing/putting away dishes	38
Toileting/showering	82
Doing/putting away laundry	34
Cleaning	10
(b) van Kasteren Dataset	
Leave house	52
Toileting/showering	153
Go to bed	31
Preparing meal/beverages	83

**Table 2:** Results of the proposed method based on tree alignment.

Trees	$\tau_{1}$	$\tau_{2}$	$\tau_{3}$	$\tau_{4}$	$\tau_{5}$	$\tau_{6}$	$\tau_{7}$	$\tau_{8}$	Average Similarity Score
(a) MIT PlaceLab Dataset									
$\tau_{1}$	-	8	6	8	−3	10	−12	5	3.14
$\tau_{2}$	8	-	15	5	3	22	−6	23	10
$\tau_{3}$	6	15	-	12	10	20	−8	18	10.43
$\tau_{4}$	8	5	12	-	3	7	−6	5	4.86
$\tau_{5}$	−3	3	10	3	-	5	4	6	4
$\tau_{6}$	10	22	20	7	5	-	−7	22	**11.29**
$\tau_{7}$	−12	−6	−8	−6	4	−7	-	−6	−5.86
$\tau_{8}$	5	23	18	5	6	22	−6	-	10.43
**Trees**	$\tau_{1}$	$\tau_{2}$	$\tau_{3}$	$\tau_{4}$	$\tau_{5}$	$\tau_{6}$	**Average Similarity Score**		
(b) van Kasteren Dataset									
$\tau_{1}$	-	14	−4	12	21	−2	**8.2**		
$\tau_{2}$	14	-	0	16	14	−3	**8.2**		
$\tau_{3}$	−4	0	-	2	−4	−12	−3.6		
$\tau_{4}$	12	16	2	-	12	−4	7.6		
$\tau_{5}$	21	14	−4	12	-	2	**8.2**		
$\tau_{6}$	−2	−3	−12	−4	−2	-	−4.6		

**Table 3:** Recognition accuracy of decision trees trained on different test sets.

Test Sets	$\tau_{1}$	$\tau_{2}$	$\tau_{3}$	$\tau_{4}$	$\tau_{5}$	$\tau_{6}$	$\tau_{7}$	$\tau_{8}$
(a) MIT PlaceLab Dataset								
1st Set	76.9	92.3	94.2	84.6	92.3	96.2	96.2	96.2
2nd Set	91.9	91.9	94.6	94.6	94.6	94.6	100	94.6
3rd Set	97.1	97.1	91.2	97.1	94.1	91.2	91.2	91.2
4th Set	93.9	95.9	98	87.8	98	98	100	98
5th Set	97	93.9	93.9	97	84.8	93.9	93.9	93.9
6th Set	95	90	100	95	95	90	95	100
7th Set	96.3	94.4	92.6	94.4	94.4	92.6	88.9	92.6
8th Set	87.1	90.3	83.9	90.3	93.5	87.1	90.3	83.9
**Average**	91.9	93.2	93.5	92.6	93.4	92.9	**94.4**	93.8
**Test Sets**	$\tau_{1}$	$\tau_{2}$	$\tau_{3}$	$\tau_{4}$	$\tau_{5}$	$\tau_{6}$		
(b) van Kasteren Dataset								
1st Set	100	100	100	100	100	100		
2nd Set	97.5	96.3	97.5	98.8	97.5	97.5		
3rd Set	98.1	98.1	84.9	98.1	98.1	98.1		
4th Set	100	100	100	100	100	100		
5th Set	96.8	96.8	96.8	93.5	96.8	93.5		
6th Set	96.9	95.3	95.3	95.3	96.9	90.6		
**Average**	**98.2**	97.7	95.7	97.3	**98.2**	96.6		

**Table 4:** Computational performance (in sec) between our method and the baseline methods. The results are the average of 30 runs on each test set.

Test Sets	Baseline Method		Proposed Method
	Naive Approach	Wrapper Method	
(a) MIT PlaceLab Dataset			
1st Set	0.03	2.53	0.05
2nd Set	0.04	2.42	0.03
3rd Set	0.03	2.46	0.03
4th Set	0.03	2.43	0.03
5th Set	0.03	2.48	0.03
6th Set	0.03	2.45	0.03
7th Set	0.03	2.38	0.03
8th Set	0.04	2.55	0.03
(b) van Kasteren Dataset			
1st Set	0.03	1.72	0.01
2nd Set	0.03	1.78	0.01
3rd Set	0.03	1.73	0.01
4th Set	0.03	1.72	0.01
5th Set	0.03	1.73	0.01
6th Set	0.03	1.68	0.01

**Table 5:** Number of sensors selected by each method.

Methods	MIT PlaceLab	Van Kasteren
Full Set of Baseline Sensors	24	14
Baseline Method—A Naive Approach	16	6
Baseline Method—Wrapper with Sequential Forward Selection	20	7
Proposed Method	13	6
